# Making van der Waals Heterostructures Assembly Accessible to Everyone

**DOI:** 10.3390/nano10112305

**Published:** 2020-11-21

**Authors:** Sergey G. Martanov, Natalia K. Zhurbina, Mikhail V. Pugachev, Aliaksandr I. Duleba, Mark A. Akmaev, Vasilii V. Belykh, Aleksandr Y. Kuntsevich

**Affiliations:** 1 National Research University Higher School of Economics, Moscow 101000, Russia; martanovsg@lebedev.ru (S.G.M.); nkzhurbina@edu.hse.ru (N.K.Z.); 2 Moscow Institute of Physics and Technology, Dolgoprudny 141701, Russia; Pugachev.MV@phystech.edu (M.V.P.); Dulebo.AI@phystech.edu (A.I.D.); 3 P.N. Lebedev Institute of the RAS, Moscow 119991, Russia; akmaevma@lebedev.ru (M.A.A.); belykh@lebedev.ru (V.V.B.)

**Keywords:** 2D materials, dry transfer, van der waals heterostructures, home-made setup, transition metal dichalcogenides, photoluminescence

## Abstract

Van-der Waals heterostructures assembled from one or few atomic layer thickness crystals are becoming increasingly more popular in condensed matter physics. These structures are assembled using transfer machines, those are based on mask aligners, probe stations or are home-made. For many laboratories it is vital to build a simple, convenient and universal transfer machine. In this paper we discuss the guiding principles for the design of such a machine, review the existing machines and demonstrate our own construction, that is powerful and fast-in-operation. All components of this machine are extremely cheap and can be easily purchased using common online retail services. Moreover, assembling a heterostructure out of exfoliated commercially available hexagonal boron nitride and tungsten diselenide crystals with a pick-up technique and using the microphotolumenescence spectra, we show well-resolved exciton and trion lines, as a results of disorder suppression in WSe${}_{2}$ monolayer. Our results thus show that technology of the two-dimensional materials and heterostructures becomes accessible to anyone.

## 1. Introduction

Two-dimensional materials and van der Waals heterostructures area is developing explosively, driving scientific breakthroughs [1,2], technology development [3,4] and device applications [5,6]. The main idea of this field is to obtain new functionality by joining atomically thin flakes or films of layered materials. For the device technology CVD [7,8], epitaxy [9], physical vapour transport (PVT) [10] and electrophoresis [11,12,13,14] methods of deposition and assembly are preferable. Most of laboratory research on van der Waals heterostructures however rely on deterministical mechanical placement of the flakes.

As a rule, the flakes for such transfer are obtained by splitting a single crystal using the “scotch tape” method, CVD or PVT growth, or by liquid phase exfoliation [15]. Atomically thin flakes most commonly are placed onto PDMS or an oxidized silicon wafer, where they are held by van der Waals forces and could be optically identified [16]. A heterostructure is then assembled out of the flakes using dry [17,18,19,20,21,22,23] or wet transfer [24,25,26,27,28], see also [3,29] for review.

Dry transfer methods have become much more common because of their convenience. They are carried out using transparent temporary substrates (TTS), sometimes called stamps. The TTS is a piece or a drop of viscoelastic transparent material, as a rule polydimethylsiloxane (PDMS), placed on the surface of the slide. The drop of PDMS is often covered with a gluing compound, usually PPC (polypropylene carbonate) [21,22] polycarbonate [19]), PMMA [17,23] or polyvinyl chloride (PVC) [30]). The adhesion of these polymers is temperature-dependent.

**Figure 1:**
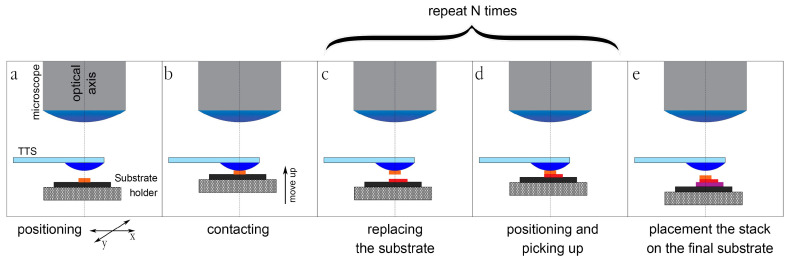
Stages of the dry pick-up transfer process for VdW heterostructure assembly.

The transfer typically proceeds as follows: The substrate with the flakes is fixed on a substrate holder (SH). It is positioned in a microscope field of view and the TTS touches a desirable flake on a substrate at a certain temperature (about 40 °C for PPC [21]) (see Figure 1a). The flake sticks to the surface of the drop. Then the drop with the flake is lifted (see Figure 1b). Then the substrate is replaced with the next one containing the other flake (see Figure 1c). The operator positions the TTS in a way that the previously collected flake overlaps with a new desired one (see Figure 1d) and picks it up. These operations (Figure 1c,d) are repeated several times until a whole stack is collected on the drop. Then the stack is transferred onto a final substrate (see Figure 1e). The temperature is set high to make PPC more liquid, the droplet is raised and the sample peels off, remaining on a substrate surface.

To carry out these heating and positioning operations, a transfer machine is required. Mask aligners, e.g., like in ref. [31] have all necessary positioning functions. There are also several reports of home-made machines [17,19,32,33,34,35,36] from the simplest for 1 k$ [33] with limited functionality, to fully robotic for 1 M$ [34]. Transfer machines are now produced commercially, e.g., by www.hqgraphene.com and www.creativedevices.com. Laboratories are often faced the task of building such a machine within quite a limited budget and time. Despite several reviews on the transfer processes [3,29,37], we did not find a description of the basic guiding opto-electro-mechanical principles those may help to avoid mistakes and assemble a powerful and cheap machine. This paper aims to fill this gap.

Another important component of van der Waals heterostructures technology is using the proper materials, as a rule exfoliated single crystals or CVD/PVT materials on substrates. These materials could be grown (the most expensive route) or are available to institutions or also could be purchased from e.g., www.hqgraphene.com, www.2dsemiconductors.com, graphene-supermarket.com. Recently there also appeared 2D material online shops on Aliexpress platform. A key element for most of heterostructures—hexagonal BN was mostly supplied by NIMS (Tsukuba) [38]. In our paper we show, by means of photoluminescence, that the crystals from Aliexpress also work well, at least in sense of interface with WSe${}_{2}$. Thus, we demonstrate that the entry threshold for van der Waals heterostructures technology becomes vanishing both for laboratories and individuals.

## 2. Design of the Transfer Machine: Guiding Principles

**Figure 2:**
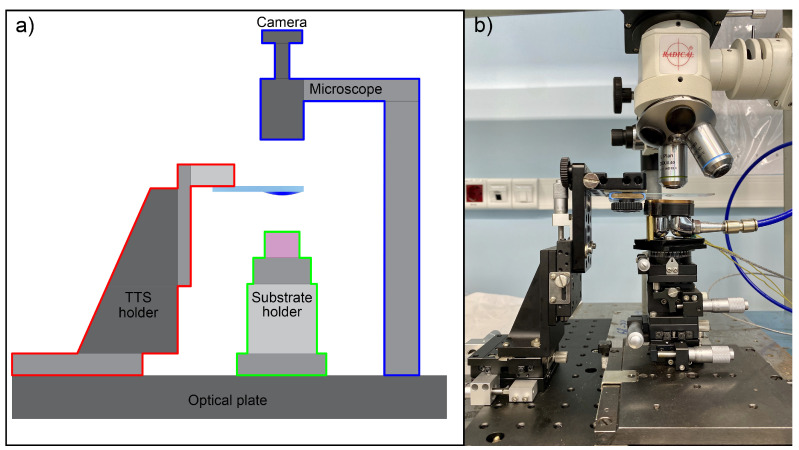
(**a**) Schematics of the transfer machine; (**b**) photo of our machine.

Any transfer machine should necessarily contain three main components, shown in Figure 2a:

1. Substrate holder
2. TTS holder
3. Microscope

We describe below the requirements to each of them using our own home-made setup as an example. Note that for a better focusing, the mutual vibrations motion of the microscope, TTS and SH should be minimized. A straightforward way is attachment of all components to a single base, e.g., an optical plate with screwholes. Such placement allows relocation of the whole setup into the glovebox to operate with materials those are unstable at ambient conditions, similarly to ref. [36]. We used a 200 × 300 mm${}^{2}$ anodized aluminum plate from www.aliexpress.com with an array of M6 screw holes equipped with rubber legs.

### 2.1. Microscope

The operator needs to have a view of the working area (typically 500 × 500 μm${}^{2}$−20x Plan objective) during the transfer process. Therefore, the device must contain a microscope. To find the flakes on a substrate an objective with smaller magnification is needed (field of view more than 2 × 2 mm${}^{2}$−5x Plan objective). It is also desirable to inspect the wafers with high magnification (field of view below 100 × 100 μm${}^{2}$−50x Plan Objective). The objectives should have large working distance (more than 4 mm) to avoid touching the glass and should be placed on the revolver head. Exactly this set of objectives is used in the top class laboratory [35,39] and commercial [www.hqgraphene.com] transfer machines. Note that the high-magnification objective is useless for transfer, because its depth of field is too small, that makes it impossible to have all field of view focused. Moreover searching for focus is time consuming with high-magnification objective. We also note that more expensive Plan Achromatic and Apochromatic objectives could possibly be useful for inspection (50x) purpose, but are excess for transfer. For detailed review on objectives see tutorials from Zeiss website and Nikon website. The microscope should be equipped with fast (more than 30 fps) and high resolution (more than 20 MP) camera for real-time monitoring and taking photos of all stages of the process. Such a camera might be puchased on www.aliexpress.com for less than $100, it can be connected to PC or monitor. Binoculars are convenient and very useful for adjustment purpose, however they hardly could be applied in the glovebox. Since the substrate is generally not transparent, the illumination should be fed through the objective.

Most of high-magnification microscopes have stationary objectives attached to a heavy base. However, for transfer machines it is necessary to move the TTS and the substrate holder with respect to each other focusing alternately on both of them. It is vital therefore to have objective height adjustable. There are commercially available microscopes with the revolver head and objective movable in z−direction e.g., by www.zeiss.com, www.olympus-ims.com and www.mitutoyo.com. They are superior in quality and pretty expensive (several 10 k$). Recently much cheaper microscopes appeared: ICM-100 (China, 1.5 k$, available from www.aliexpress.com) and Radical (India, $400, available from www.amazon.com). 

The last one was used in our setup. Besides the price its advantage is a small size, that allows a subsequent placement into the glove-box, similarly to ref. [36]. Large working-distance objectives for this microscope were purchased separately on www.aliexpress.com. A disadvantage of this microscope is mismatching of visible areas in different slots of objective turret.

A microscope with a single objective and zoom, easily available on www.aliexpress.com, may also be considered as an option, as in ref. [33]. Note that zoom adjustment automatically de-focus the microscope.

### 2.2. Transparent Temporary Substrate Holder

The function of this part is to hold the glass slide with a drop/stamp. The glass should be fixed by the clips in the space between the sample and the microscope. Atomically thin flakes are practically invisible on the TTS, therefore, when assembling the stacks, the microscope optical axis and the TTS holder should remain mutually immobile, otherwise the stack on the TTS could be lost. At the same time the substrates with flakes should be movable and easily changeable. Before the transfer, the lowest point of the PDMS drop should be located in the microscope field of view.

Thus, the TTS holder should have at least two degrees of freedom along the *x* and *y* axes. If the microscope has a stationary objective, then the TTS holder must also have *z*-degree of freedom. There are many options for the design of the TTS holder. For example, a manipulator from the probe-station might be used for this purpose [21], that can be purchased on www.aliexpress.com.

We have used XYZ-linear stage from www.aliexpress.com and attached a glass slide using the cheeseplates for photography, also purchased on www.aliexpress.com.

**Figure 3:**
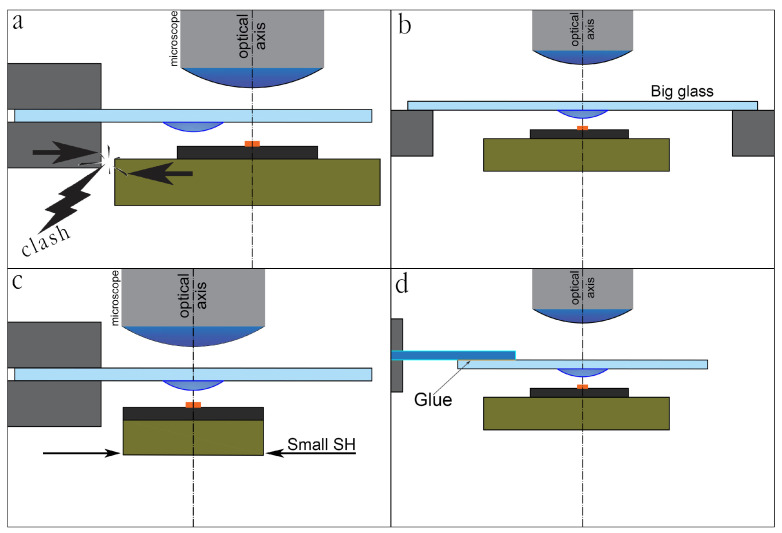
Problem of too small glass slide (**a**) and its possible solutions: (**b**) using large glass e.g., like in mask aligner; (**c**) making a small sample holder table; (**d**) gluing a glass slide or stamp to the end of the other slide.

The main challenge with the TTS holder is illustrated in Figure 3a. Standard glass slides are rather short (≈76 mm). The drop should be located in the center of the glass to spin-coat it with PPC. Typically the radius of the SH table exceeds half of the glass slide length (∼3 cm), and the table touches the clamp, that is not acceptable. There are several approaches to overcome this issue, illustrated in Figure 3b–d. If a glass slide is large, it can be safely positioned similarly to mask-aligner based machines [31] (Figure 3b). The table size could also be minimized (Figure 3c), for example in our machine the diameter of the table is less than 6 cm, so the TTS might fit the center of the SH. Most commonly, the stamp is either relocated to the end of the glass slide or to another glass slide [35,39] that is glued to the bottom of the holder, as shown in Figure 3d. The combination of these approaches is also possible.

### 2.3. Substrate Holder

Substrate holder should allow positioning with μm resolution along the *x*,*y*, and *z* directions. For creating the twisted structures [32,40] it is also necessary to rotate the substrate in the horizontal plane with a fractions of a degree precision. Corresponding manipulators are available commercially and relatively cheap (∼$150 on www.aliexpress.com ).

Note that all manual motion and rotation stages could be upgraded to the motorized ones, that is useful for glovebox operation and helps to avoid vibrations [35].

It is convenient to remove the substrate holder, replace the substrate and return the holder to exactly the same place without displacement of the stack on the TTS. In ref. [33] a SH with magnetic base was used for this purpose. It is more reproducible to place the manipulator on a single-axis slide with a locking screw. Such a slide is available on www.aliexpress.com and allows to change a substrate in a few seconds (see Supplementary VideoADD HYPERREFERENCE TO SUPPLEMENTARY VIDEO).

The substrate is placed on the table, that is attached to the manipulator. The dry transfer pick-up process is based on sensitivity of the polymer to temperature. So the table should contain a heating element and a thermometer. It is also necessary to seal the substrate. Entry-level solution is fixing the substrate with glue or double-sided scotch-tape. The obvious disadvantages are contamination of the substrate backside, and in the case of glue, a long waiting time. Moreover, the glue might lose the adhesive properties with temperature. The fastest and most convenient sealing is vacuum suction of the substrate.

The heating table should therefore have the following components:

1. A vacuumed holder with metallic polished top plate drilled for vacuum fixation of the the substrate.
2. A heater to change the sample temperature.
3. A thermocouple to control these changes.

**Figure 4:**
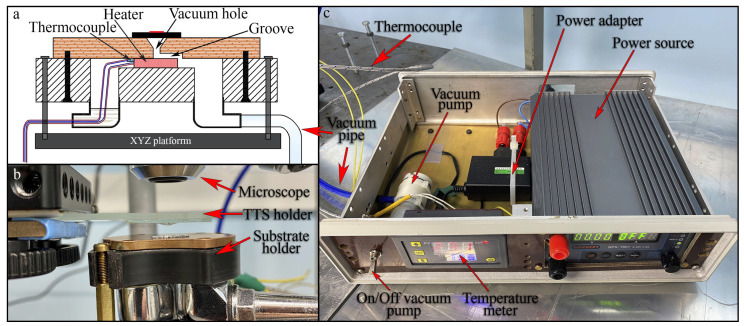
(**a**) Schematics of the substrate holder (**b**) photo of the substrate holder, (**c**) photo of the electronic box.

Commercially available heated vacuum chucks are expensive and massive, see e.g., solutions by www.tceramics.com and www.thermocoax.com. Making a customised one in a mechanic workshop from the sketch might be time consuming. We built a cheap and compact one out of PC Motherboard Bridge waterblock from www.aliexpress.com as shown in Figure 4a,b.

We place a heater (from www.aliexpress.com) and a thermocouple (from www.aliexpress.com) inside a chamber of the waterblock and drill a hole in the center of the copper plate for vacuum sealing of the substrate. Heater and thermocouple wires pass through one of the water entrances. In order to make a path for vacuum above the heater a groove was milled to the pumping hole, as shown in Figure 4a. The tube for vacuum pumping passes through the second water entrance.

Two hermetically sealed 90° adaptors (from www.aliexpress.com) are used to feed the wires and pumping line horizontally.

All electronic components for the table operation are placed into a box shown in Figure 4c. We take a cheap (∼$3 on www.aliexpress.com) aquarium diaphragm pump for vacuum and a standard PID-controller from www.aliexpress.com to measure the temperature. One common mobile phone AC/DC adaptor (5 V, 2 A) is used for both pump and temperature meter. The pump has a switch on the front panel. A laboratory power source (0–32 V, 0–5 A by Gophert, available from www.aliexpress.com) is used for the heater instead of PID-controller, since the latter appeared to be too slow.

Using a computer control similarly to www.hqgraphene.com machine probably makes the process faster, more controllable and makes sense if the mass production of the van der Waals heterostructures is needed. Such automatization is not required for a small scientific group with limited measurement facilities where the bottleneck is time-consuming TTS preparation, material exfoliation and search.

## 3. Examples of Operation

**Figure 5:**
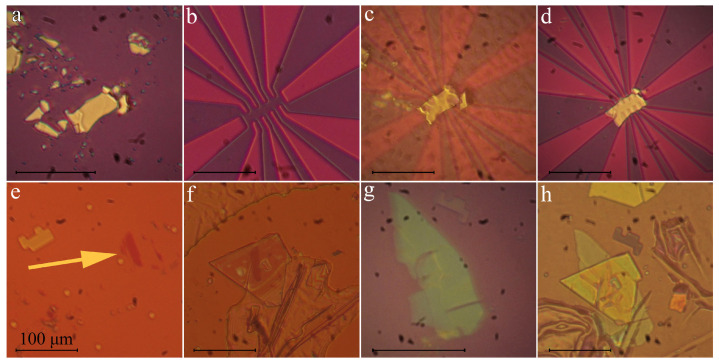
(**a–d**) Transfer process of Sr${}_{x}$Bi${}_{2}$Se${}_{3}$ to prepared gold contacts (**e–h**) Stacking process for heterostructure hBN-WSe${}_{2}$-hBN.

Figure 5 shows examples of the systems assembled with the aid of our setup using the standard PPC on PDMS drop route [21]. An approximately 50 nm thick flake of Sr${}_{x}$Bi${}_{2}$Se${}_{3}$ (see Figure 5a) was obtained by scotch tape exfoliation onto an oxidized Si wafer. The growth and characterization of Sr${}_{x}$Bi${}_{2}$Se${}_{3}$ single crystals are described in refs. [41,42]. Contact electrodes were lithographically defined on an oxidized Si wafer using a laser pattern generator Heidelberg μPG101. 30 nm thick gold conducting layer was thermally evaporated and a standard lift-off process was performed (see Figure 5b). We locate the flake through the TTS, pick it up, change a substrate and find the contact electrodes, align the contacts and the flake (Figure 5c) and peel the flake off (Figure 5d). The whole transfer process takes about 7 min.

Figure 5e–h shows a van der Waals heterostructure boron nitride/monolayer WSe${}_{2}$/boron nitride. We used commercially available hexagonal boron nitride from www.aliexpress.com and thungsten diselenide from www.aliexpress.com. Boron nitride was scotch-tape exfoliated onto an oxidized Si wafer (285 nm oxide thickness) from www.aliexpress.com. We isolated the WSe${}_{2}$ monolayer using the gold layer, similarly to ref. [43]. In order to simplify the micro-photoluminescence experiments we transfer the flakes onto an array of markers. Figure 5 shows all elements and the assembly. The whole transfer process took about 12 min. Note that the markers are also required if the beam lithography operations will follow.

In the Supplementary Video ADD HYPERREFERENCE TO VIDEO we show the example of the pick-up and transfer process.

**Figure 6:**
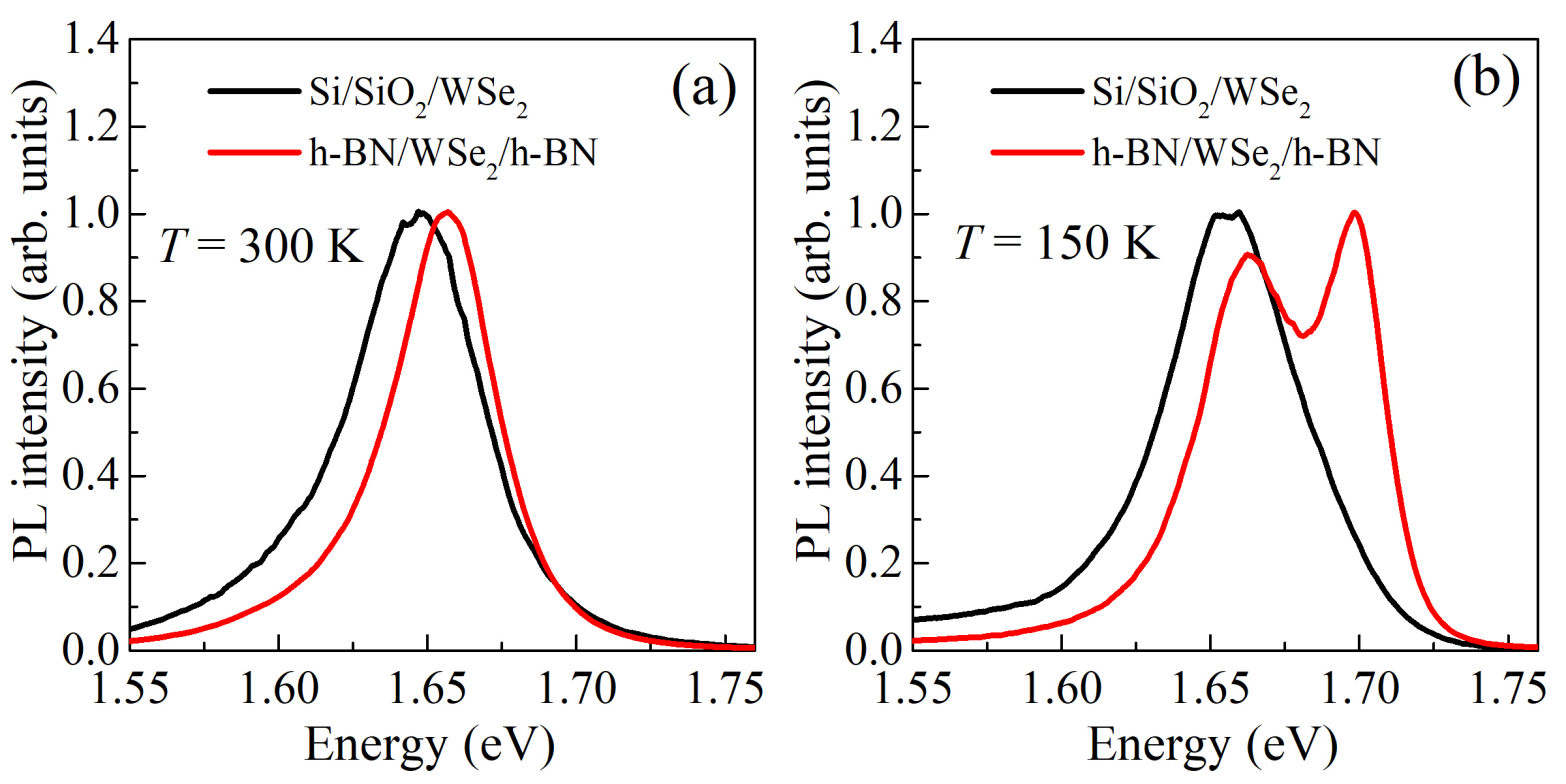
Comparison of the photoluminescence spectra for non-encapsulated and encapsulated WSe${}_{2}$ monolayers at room (**a**) and low (**b**) temperatures. The spectra are normalized to maximum.

To demonstrate the quality of thus fast-assembled heterostructures with monolayer WSe${}_{2}$ we used micro-photoluminescense (PL) as a common tool. The sample was excited by the emission of a diode laser operating at wavelength of 457 nm. The laser beam was focused on a sample onto a spot of 2 μm size using a Mitutoyo microobjective with large working distance. The same microobjective was used to observe the sample surface with a video-camera and to collect PL. The PL spectra were measured using a spectrometer with a nitrogen-cooled CCD detector. In Figure 6 we compare micro-PL spectra collected from encapsulated and non-encapsulated WSe${}_{2}$ monolayers at room and low temperatures. At room temperature (Figure 6a) the position of exciton emission line in the encapsulated sample is shifted due to difference in dielectric constants and its linewidth decreases due to disorder suppression. At lower temperature of 150 K neutral and charged exciton lines (higher and lower in energy, respectively) become well-distinguishable for the heterostructure. The neutral exciton linewidth of about 22 meV, is partly contributed by the thermal broadening $k_{B}T\approx 13$ meV, and demonstrates a feasible improvement of the quality of the structure.

## 4. Discussion

It should be noted that the suggested machine uses all the design principles implemented in the top-level transfer machines, such as:

1. Independent and precise positioning of all three components of the machine (microscope, TTS holder, substrate holder);
2. Presence of microobjectives with different magnification for transfer, location and analysis;
3. Vacuum chuck and temperature control on the substrate holder.

Moreover this design has some advantages:

1. Compact size for easy integration into a glovebox;
2. Low cost, about 1 k$ for all components;
3. Modular design of the device;
4. Cheap and compact substrate holder table;
5. Convenient substrate replacement without touching the TTS holder and the microscope.

Some other technical aspects of the transfer machine should also be discussed:

- Vacuum sealing of the substrate is never perfect, there is some air flow under the substrate that cools the sample down making a thermometer misleading. This effect should be taken into account.
- It is highly desirable not to lose a focus during the substrate positioning and rotation. Therefore the sample holder table plane and microscope plane of view should be parallel and aligned perpendicularly to the rotational axis of the holder. The later axis should be rather close to the optical axis, otherwise it will take extra time to find the flakes after the rotation.
- The construction by www.hqgraphene.com is a bit different from the one considered in this paper as its xy-manipulator (TTS Holder) is placed onto another bigger manipulator (substrate holder). Such a placement is fruitful idea, as it suppresses mutual vibrations of the substrate and the TTS and can help to avoid extra degrees of freedom.
- Note that the machine is assembled out of very cheap components because there is no need in sub-micrometer resolution positioning. For our machine we estimate the precision of flakes alignment as 2 micrometers, that comes from diffraction limit of the microscope, vibrations and viscoelastic properties of PDMS, those lead to slight shift of flakes in the process of TTS-to-substrate contact.
- The transfer with the optimized machine might be very fast and takes just a few minutes provided that the TTS is well prepared. One drop of PDMS might be used many times. However, sometimes the transfer might be unsuccessful. This is because there are hidden controlling parameters those crucially affect the properties of the thermosensitive polymer, such as humidity, thickness of the polymer layer, freshness of the PDMS drop, preparation of the substrate surface etc. These parameters determine the optimal temperature regime and should be maintained stable for a reproducible process. Searching for flakes and controlling these parameters takes much more time than the transfer itself.
- In refs. [17,23] a Peltier element, easily available from www.aliexpress.com, is used instead of resistive heater. This element is faster, though it is not compatible with vacuum fixation, and the authors have to use the double-sided scotch-tape. The temperature range for the Peltier element is also smaller (between 10 °C and 80 °C).
- Concerning the price. Similarly to our setup ref. [33] reports 1 k$ transfer machine. That machine however lacks for temperature-controlled table and therefore does not allow pick-up process. We believe that this price is close to absolute minimum and there is no need to further optimize it, because mechanical elements could hardly be made cheaper than 400 $ and the microscope absolute minimum is close to 600 $. In fact such a price means that transfer machine, equipped with all possible degrees of freedom, becomes available for any laboratory or even person, that is the main message of this paper.

Now let us also discuss the above example with emission from the encapsulated WSe${}_{2}$ monolayer. Absence of the optimization (annealing of the layers) and using not too-high quality commercially available materials, of course, increases the PL linewidth for both encapsulated and insulated WSe${}_{2}$ monolayers. For the top quality materials, in WSe${}_{2}$ monolayers neutral exciton line narrowing from ∼13 to ∼4 meV at 4 K was reported [44]. In our paper, using materials at hand, we have demonstrated the encapsulation of the WSe${}_{2}$ in h-BN that allows to resolve different exciton states that were not resolved for insulated WSe${}_{2}$. It is already sufficient to address these states separately and perform further optical investigation on exciton physics in monolayers.

## 5. Conclusions

We demonstrate the guiding construction principles for cheap and multi-functional van der Waals heterostructures transfer machines taking our own machine as an example. We essentially exploit hot pick-up process, that allows to assemble the structures from the individually isolated monolayers. All required materials could be purchased on Amazon and AliExpress. This paper thus helps those who want to enter the 2D materials and van der Waals heterostructures field with maximal outcome per minimal expenses.

## Supplementary Materials

The following are available online at https://www.mdpi.com/2079-4991/10/11/2305/s1.

## Abbreviations

The following abbreviations are used in this manuscript:

SH	Substrate holder
TTS	Transparent temporay substrate
PVA	Polyvinylalcohol
CVD	chemical vapour deposition
PPC	Poly-propilene carbonate
PID	Proportional-integral-differential (controller)
PVC	poly-vinyl chloride
PMMA	poly-methyl meta acrylate
PC	personal computer
PL	photoluminescence
NIMS	National Institute of Materials Science in Tsukuba, Japan
